# The outcome and risk factors associated with central and peripheral nervous system involvement in hospitalized COVID-19 patients: a retrospective cohort study

**DOI:** 10.3389/fneur.2023.1338593

**Published:** 2024-01-11

**Authors:** Andreea Raluca Hanganu, Cristian-Mihail Niculae, Adriana Octaviana Dulămea, Emanuel Moisă, Rareș Constantin, Georgiana Neagu, Adriana Hristea

**Affiliations:** ^1^Faculty of Medicine, University of Medicine and Pharmacy “Carol Davila”, Bucharest, Romania; ^2^National Institute for Infectious Diseases “Prof. Dr. Matei Bals”, Bucharest, Romania; ^3^Fundeni Clinical Institute, Bucharest, Romania; ^4^Elias University Emergency Hospital, Bucharest, Romania

**Keywords:** COVID-19, SARS-CoV-2, peripheral nervous system, risk factors, analysis, central nervous system, outcome

## Abstract

**Introduction:**

SARS-CoV-2 infection can affect any organ, including both the central nervous system (CNS) and peripheral nervous system (PNS). The aim of this study was to explore the outcome and risk factors associated with the involvement of either CNS or PNS in a cohort of hospitalized COVID-19 patients.

**Methods:**

We performed a retrospective observational cohort study of hospitalized adult patients with COVID-19, between May 2020 and December 2022, presenting with new onset neurological disabilities any time after admission.

**Results:**

We included 115 patients, 72 with CNS manifestations and 43 with PNS involvement. The CNS manifestations were COVID-19-associated encephalopathy, headache, neurovascular events, and seizures in 80.5, 43, 31.9, and 11.1% of patients, respectively. The neurovascular events were ischemic stroke in 17 (23.6%) patients, hemorrhagic stroke in 6 (8.3%) patients, venous thrombosis in 1 (1.4%) patient, and subarachnoid hemorrhage in 1 (1.4%) patient. Cranial nerve involvement was the most frequent PNS manifestation in 34 (79%) cases, followed by mononeuritis in 5 (11.6%) patients and polyneuropathy in 4 (9.3%) patients. The affected cranial nerves were the vestibulocochlear nerve in 26 (60.5%) patients, the olfactory nerve in 24 (55.8%) patients, the oculomotor nerves in 5 (11.6%) patients, and the facial nerve in 1 (2.3%) patient. Two patients (9.3%) presented with polyneuritis cranialis. Older age (HR = 1.02, 95% CI: 1.003–1.037, *p* = 0.01), COVID severity (HR = 2.53, 95% CI: 1.42–4.5, *p* = 0.002), ischemic cardiac disease (HR = 2.42, 95% CI: 1.05–5.6, *p* = 0.03), and increased D-dimers (HR = 1.00, 95% CI: 1.00–1.00, *p* = 0.02) were independently associated with the development of CNS manifestations. The factors associated with in-hospital mortality were age (HR = 1.059, 95% CI: 1.024–1.096, *p* = 0.001), C-reactive protein (HR = 1.006, 95% CI: 1.00–1.011, *p* = 0.03), CNS involvement (HR = 9.155, 95% CI: 1.185–70.74, *p* = 0.03), and leucocyte number (HR = 1.053, 95% CI: 1.026–1.081, *p* < 0.001).

**Conclusion:**

COVID-19-associated encephalopathy was the most common CNS manifestation in our study, but neurovascular events are also important considering the overlap between inflammatory and prothrombotic pathways, especially in severe cases. CNS involvement was associated with in-hospital all-cause mortality. PNS findings were various, involving mostly the cranial nerves, especially the vestibulocochlear nerve.

## 1 Introduction

COVID-19 is a complex respiratory and systemic disease, with a variable range of severity. Both respiratory and non-pulmonary complications can occur as SARS-CoV-2 has a broad tissue tropism. Neuropilin-1 (NRP-1) facilitates its entry into the central nervous system (CNS) through the olfactory epithelium of the nasal cavity (1). Neurological deficits are one of the most difficult to prevent and manage complications, and they usually occur in severe forms of disease (2, 3). They can range from mild symptoms (anosmia, dysgeusia, etc.) to more severe complications, such as encephalopathy, stroke, cerebral venous thrombosis, seizures, and Guillain-Barre syndrome (GBS), influencing the general outcome of the patients (3–5). The pathophysiology behind these complications, as well as the associated risk factors and optimal management, is still a subject of research in the medical community (6).

However, some studies reported only on the prevalence of the neurological manifestations in COVID-19 (7), included patients with preexisting neurological conditions (8), or reported only on the outcome of patients with COVID-19 and some of the most common neurological manifestations (7, 9). Therefore, reports on risk factors associated with either peripheral nervous system (PNS) or CNS manifestations in COVID-19 are limited.

The aim of this study was to describe new onset CNS and PNS involvement during COVID-19 in patients requiring hospitalization and to explore the risk factors associated with either CNS or PNS disabilities. In addition, we analyzed the outcomes of these patients to identify patient characteristics associated with unfavorable outcomes.

## 2 Materials and methods

### 2.1 Study design and participants

**Figure 1:**
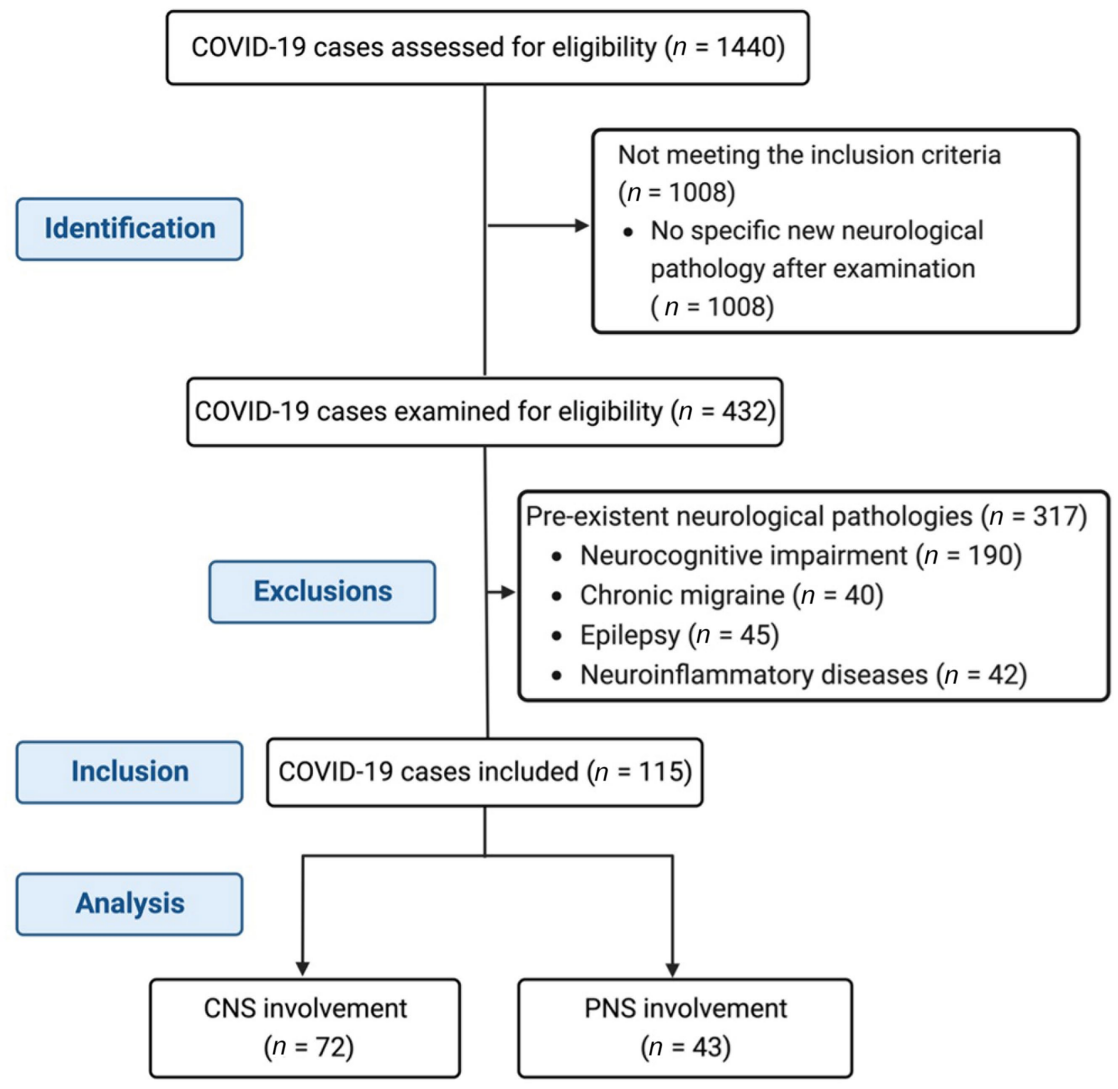
Flowchart of the study: recruitments, inclusions, and exclusions.

We performed a retrospective observational cohort study that included hospitalized patients with COVID-19, between May 2020 and December 2022. We included adult patients (over 18 years old) who developed new-onset neurological symptoms during hospitalization. Only patients with confirmed SARS-CoV-2 infection who had a clinical examination performed by a neurologist were assessed for eligibility. They were selected from the specific medical records with a neurological examination, performed when clinically indicated. All patients were examined by the same local neurologist established to care exclusively for COVID-19 patients in our infectious disease tertiary facility. We excluded patients with preexistent neurological chronic diseases, such as neurocognitive impairment, chronic migraine, epilepsy, and neuroinflammatory disorders. The flowchart of the study is presented in Figure 1. This study was approved by the Local Bioethics Committee.

### 2.2 Data collection

We reviewed electronic and physical medical records of the patients, which included demographic data (age and gender), clinical data on comorbidities (high blood pressure, atrial fibrillation, ischemic heart disease, stroke history, atherosclerosis, diabetes mellitus, obesity, and chronic kidney disease), lifestyle, severity of COVID-19 and respiratory parameters, laboratory data (complete blood count, C-reactive protein—CRP, ferritin, creatinine, urea, liver enzymes, erythrocyte sedimentation rate, D-dimers, fibrinogen, lactate dehydrogenase—LDH, sodium, potassium, glycemia, and other metabolic parameters), and pulmonary and brain imaging data (head CT scan and/or MRI scan). No follow-up data post-hospitalization were available.

### 2.3 Definitions

Neurological data were extracted from the specific clinical examinations and diagnosis made by the neurologist, and it was categorized into CNS involvement (encephalopathy, headache, neurovascular events, and seizures) and PNS findings (cranial nerve involvement, polyneuropathies, and mononeuritis). COVID-19-associated encephalopathy was defined as new onset neurocognitive decline and/or state of consciousness alteration when no other causes were identified, especially related to hypoxemia, iatrogenic, common metabolic causes, or intensive care unit admission (ICU)-related; this entity is considered to be caused by cytokine-immune-mediated inflammation (10). Unusual new headache phenotypes associated with COVID-19 were considered CNS involvement, as described by other studies, and not related to fever, hypoxemia, or other common causes at the moment of the clinical examination (11, 12). We defined the severity of SARS-CoV-2 as mild (normal oxygenation and no pulmonary lesions), medium (pneumonia on chest CT), or severe (based on at least one of the following additional criteria: peripheral oxygen saturation ≤ 93% in ambient air, respiratory rate > 30/min, arterial oxygenation partial pressure to fractional inspired oxygen ratio < 300, or lung infiltrates >50% of lung parenchyma), using the World Health Organization COVID-19 severity classification criteria as they are usually referred to in research (13).

### 2.4 Statistical analysis

Descriptive data were expressed as frequencies (%) for categorical data, means ± standard deviation (SD) for continuous variables with normal distribution, and median (range) for continuous variables without normal distribution. Normal distribution was checked using the Shapiro–Wilk test. Continuous variables were compared using the Mann–Whitney U-test for data that significantly deviated from a normal distribution. A paired-sample *t*-test was used for normally distributed continuous variables. Categorical variables were compared among groups using the chi-square test (or Fisher’s exact test if needed).

Cox proportional hazard (PH) regression was used to study the independent value of different variables regarding two outcomes during hospitalization: (1) factors associated with the development of CNS/PNS manifestations and (2) unfavorable outcomes (deceased patients). A stepwise (forward likelihood ratio) method was used for variable selection in the final model. The factors were retained in the model if *p* < 0.05 and excluded if *p* > 0.1. The results were expressed as hazard ratio (HR) with a 95% confidence interval (95% CI). All the confounding variables introduced in the regression will be available as Supplementary material for each model depending on the studied outcome. All tests were two-tailed and were considered significant if *p* < 0.05. The rationale for introducing these variables in the model was based on the basic inference analysis but also on clinical judgment and the results reported in the literature (factors independently associated with the studied outcome). For the present study, statistical analysis was performed using Statistical Package for Social Sciences (SPSS version 28, IBM Corp., Armonk, NY, United States).

## 3 Results

### 3.1 Baseline characteristics of patients with COVID-19 and neurological symptoms

During the studied period, from all hospitalized patients, 1,440 were examined for eligibility, based on a specific clinical examination made by the neurologist. Of these, we included 115 patients, 72 with CNS manifestations and 43 with PNS involvement. The cohort was composed of 55 (47.8%) men. The mean age for the whole group was 61.7 ± 15.46 years. The median time for neurological symptoms since COVID-19 diagnosis was 9 (0–129) days, and the median hospitalization duration was 20 (2–139) days. Sixty-two (53.9%) patients had severe COVID-19. In terms of cardiovascular risk factors, 66 (57.4%) patients had high blood pressure, 40 (34.8%) patients had diabetes mellitus, 18 (15.7%) patients had atrial fibrillation, 11 (9.6%) patients had ischemic heart disease, 25 (21.7%) patients had atherosclerosis, 33 (28.7) patients had obesity, 7 (6%) patients had chronic kidney disease, 4 (3.5%) patients had patients were smokers, and 1 (0.8%) patient suffered from unhealthy alcohol use. Inflammatory markers such as CRP, ferritin, and fibrinogen had means (±SD) of 78.7 (±82.7), 749.5 (±574.7), and 491.1 (±189.2), respectively. Mean values for D-dimers, INR, and LDH were 816.3 (±2,825), 1.17 (±0.04), and 452.55 (±330.8), respectively.

In Table 1, the comparative demographics and characteristics of patients with CNS and PNS involvement are summarized. Compared to patients who presented with PNS manifestations, patients who had CNS involvement were older, predominantly males, had more immunosuppressive conditions such as diabetes mellitus and chronic kidney disease, and had severe forms of SARS-CoV-2 infection. The onset of CNS neurological symptoms was more likely to develop later in the course of the COVID-19 vs. PNS manifestations.

**Table 1:** Clinical and laboratory data of patients with CNS and PNS involvement associated with COVID-19.

		CNS involvement *n* = 72	PNS involvement *n* = 43	*p* OR (95 CI)
Male, *n* (%)		40 (55.6)	14 (32.6)	0.02 2.6 (1.2-5.7)
Age, median (range)		68 (32-92)	56 (25-88)	<0.001 10.75 (5.2-16.3)
Time to neurological symptoms (days), median (range)		11 (0-67)	5 (0-129)	0.3 3 (-3.8-9.9)
Severe COVID, *n* (%)		46 (63.9)	16 (37.2)	0.007 0.3 (0.1-0.7)
Hospitalization duration (days), median (range)		25.5 (2-115)	15 (3-139)	0.5 5.2 (-7.1-13.5)
**CVD risk factors**, *n* (%)	High blood pressure	46 (63.9)	20 (46.5)	0.08 0.5 (0.2-1.1)
	Diabetes mellitus	32 (44.4)	8 (18.6)	0.005 0.3 (0.1-0.7)
	Atrial fibrillation	14 (19.4)	4 (9.3)	0.1 0.4 (0.1-1.4)
	Ischemic cardiac disease	8 (11.1)	3 (7.0)	0.5 0.6 (0.1-2.4)
	Stroke history	6 (8.3)	4 (9.3)	0.5 1.1 (0.3-4.2)
	Atherosclerosis	17 (23.6)	8 (18.6)	0.6 0.7 (0.3-1.9)
	Obesity	22 (30.6)	11 (25.6)	0.6 0.8 (0.3-1.8)
	Chronic kidney disease	7 (9.7)	0 (0)	0.04 0.6 (0.5-0.7)
	Smoker	3 (4.2)	1 (2.3)	1 0.5 (0.05-5.4)
	Alcohol consumption	1 (1.4)	0 (0)	1 0.6 (0.5-0.7)
**Laboratory data** (median ±SD)	CRP (mg/dl) (n = 114)	95 ± 88	51 ± 65	0.005 44.5 (13.7-75.4)
	Ferritin (ng/ml) (*n* = 92)	81 ± 546	633 ± 612	0.1 185.5 (-59.4-430.4)
	Fibrinogen (mg/dl) (*n* = 113)	526 ± 190	430 ± 174	0.009 96.3 (24.8-167.6)
	D-dimers (ng/ml) (*n* = 107)	1,051 ± 3,511	406 ± 502	0.2 645.5 (-478.1-1769)
	INR (n = 114)	1.2 ± 0.4	1.09 ± 0.1	0.1 0.1 (-0.03-0.2)
	LDH (units/L) (*n* = 111)	480 ± 335	406 ± 322	0.2 74.3 (-54.5-203)
			
**Deceased**, *n* (%)		23 (31.9)	1 (4.2)	<0.001 0.05 (0.007-0.4)

### 3.2 CNS and PNS manifestations associated with COVID-19

The main neurological manifestations in COVID-19 patients are summarized in Table 2. Among CNS manifestations, the most common was COVID-19-associated encephalopathy. We also found neurovascular events represented by ischemic stroke in a quarter of patients followed by hemorrhagic stroke, while venous thrombosis and subarachnoid hemorrhage were diagnosed in very few patients. Of these, one patient had both ischemic stroke and subarachnoid hemorrhage, and one patient had an ischemic stroke with hemorrhagic transformation. The patient with ischemic stroke and subarachnoid hemorrhage was the youngest patient with neurovascular involvement (33 years old) and had no prior vascular risk factors.

**Table 2:** CNS and PNS manifestations associated with COVID-19.

CNS, n (%) (n = 72)	PNS, n (%) (n = 43)
At least one CNS manifestation, 72 (62.6) **COVID-19-associated encephalopathy**, 58 (80.5) **COVID-19-associated headache**, 31 (43) **At least one neurovascular event,** 23 (31.9) Ischemic, 17 (23.6) Hemorrhagic, 6 (8.3) Venous thrombosis, 1 (1.4) Subarachnoid hemorrhage, 1 (1.4) **Seizures**, 8 (11.1)	**Cranial nerves**, 34 (79) Olfactory nerve, 24 (55.8) Oculomotor nerves, 5 (11.6) Facial nerve, 1 (2.3) Vestibulocochlear nerve, 26 (60.5) Polyneuritis cranialis, 2 (4.7) **Mononeuritis**, 5 (11.6) **Polyneuropathy**, 4 (9.3)

Cranial nerve involvement was the most frequent PNS manifestation in 79% of cases, followed by mononeuritis (11.6%) and polyneuropathy (9.3%). The most affected nerve was the vestibulocochlear nerve (60.5%), followed by the olfactory nerve (55.8%), the oculomotor nerves (11.6%), and the facial nerve (2.3%). Two patients (9.3%) presented with polyneuritis cranialis.

### 3.3 Factors independently associated with CNS and/or PNS manifestations

After a stepwise Cox PH regression, the following variables were identified to be independently associated with the development of CNS manifestations during hospitalization: older age (HR = 1.02, 95% CI: 1.003–1.037, *p* = 0.01), COVID severity (HR = 2.53, 95% CI: 1.42–4.5, *p* = 0.002), ischemic cardiac disease (HR = 2.42, 95% CI: 1.05–5.6, *p* = 0.03), and increased D-dimers (HR = 1.00, 95% CI: 1.00–1.00, *p* = 0.02). The results are reported in Table 3. The full regression model is available as *Supplementary material*. The only variable excluded from the analysis was the ferritin value, given that for this variable, a high proportion of data was missing. No handling was necessary regarding missing data, given that this is not a large cohort and the D-dimer value was the only variable with 7% missing data.

**Table 3:** Final Cox PH model with the factors independently associated with CNS and/or PNS manifestations.

Variables		B	SE	Wald	df	Sig.	Exp(B)	95,0% CI for Exp(B)	
								Lower	Upper
	Age at onset	,020	,008	5,576	1	,01	1,020	1,003	1,037
	COVID severity	,929	,294	9,988	1	,002	2,532	1,423	4,505
	Ischemic cardiac disease	,886	,427	4,305	1	,03	2,426	1,050	5,602
	D-dimers	,000	,000	5,410	1	02	1,000	1,000	1,000

### 3.4 In-hospital mortality of patients with CNS and PNS manifestations associated with COVID-19

Median duration of hospitalization was 25.5 (2–115) days for patients with CNS involvement and 15 (3–139) days for patients with PNS involvement (*p* = 0.5). Among patients with neurological manifestations, older age was an independent risk factor for in-hospital mortality during hospitalization (HR 1.02, CI 95% 1–1.03, *p* = 0.02) and CNS involvement (HR 9.15, CI 1.18–70.73, *p* = 0.03), together with higher CRP (HR 1, 95% CI 1–1.01, *p* = 0.03) and higher white blood count (HR = 1.05, 95% CI 1.02-1.08, *p* < 0.001).

Stepwise Cox PH regression was used to evaluate the factors independently associated with death during hospitalization. In this regard, age (HR = 1.059, 95% CI: 1.024–1.096, *p* = 0.001), CRP (HR = 1.006, 95% CI: 1.00–1.011, p = 0.03), CNS involvement (HR = 9.155, 95% CI: 1.185–70.74, *p* = 0.03), and white blood count (HR = 1.053, 95% CI: 1.026–1.081, *p* < 0.001) were selected in the final model (Table 4). The factors excluded from analysis during stepwise selection are available as Supplementary material.

**Table 4:** Final Cox stepwise PH regression for the risk of death of patients with COVID-19 and neurological manifestations.

Variables		B	SE	Wald	df	Sig.	Exp(B)	95,0% CI for Exp(B)	
								Lower	Upper
	Age	,058	,017	11,065	1	,001	1,059	1,024	1,096
	CRP	,006	,003	4,592	1	,032	1,006	1,000	1,011
	CNS vs. PNS involvement	2,214	1,043	4,506	1	,034	9,155	1,185	70,738
	White blood count	,052	,013	14,864	1	,000	1,053	1,026	1,081

## 4 Discussion

To the best of our knowledge, this is the first study to date to make a direct comparison between factors that influence either CNS or PNS manifestations in hospitalized patients with COVID-19 and associated neurological involvement. There are studies in the literature that emphasize the fact that CNS involvement appears in older patients with more severe forms of disease, who have worse outcomes (14, 15). In our study, we demonstrate that older age together with severe forms of COVID-19 is an independent risk factor for developing CNS involvement in hospitalized patients. High D-dimers are also an independent risk factor for CNS manifestations as they are a direct measurement of the COVID-19-associated coagulopathy linked to the severe forms of disease and hyperinflammation, hypoxia-related mechanisms, and thrombi formation (16, 17). The association between high D-dimer titer and poor outcomes in severe COVID-19 has been postulated before (18), but in our study, we did not find this association. We found that ischemic heart disease history is another independent risk factor for CNS involvement in COVID-19 patients, as it reflects on a preexistent vascular pathology, probably augmenting the risk of hypoxia and coagulopathy, leading to encephalopathy, stroke, and seizures. Patients with preexisting ischemic heart disease are more likely to have severe COVID-19 disease (19). In the present study, severe COVID-19 disease was mostly seen in patients with CNS manifestations, which gives valuable insight as endothelial stabilization therapy might be taken into consideration for patients who are at risk (20).

The most common CNS manifestation in our study was COVID-19-associated encephalopathy in 80.5% of patients. It manifests as an alteration of consciousness and/or acute neurocognitive disorder. In our cohort, 48.6% of patients had alteration of consciousness ranging from somnolence to profound coma. All patients underwent at least a brain CT scan, showing no specific modifications. Some patients were also investigated by brain MRI, but only one patient presented specific brain MRI modifications consistent with hypoxic encephalopathy that is hard to attribute to COVID-19 given that he underwent a prior resuscitated cardiac arrest. These data are consistent with histopathological findings reported by another study in which brain specimens are obtained from deceased patients with COVID-19, who presented an alteration of consciousness with unremarkable CT brain scans. There were also no signs of encephalitis or other changes that could be related to the virus, except the ones consistent with hypoxic lesions (21). Neural damage in the lower brainstem might result in consciousness alteration, insomnia, and cardiorespiratory dysregulation, together with CNS signs. The involvement of the brainstem is not limited to COVID-19, as many infectious and inflammatory disorders share the same pattern (22). Two of our patients underwent lumbar puncture, but the result was unremarkable and there was no evidence of the SARS-CoV-2 RNA in the cerebrospinal fluid. Although there are literature reports of SARS-CoV-2 RNA in the cerebrospinal fluid, encephalitis is a rare cause of mental status alteration in COVID-19 patients, alteration of consciousness being most probably a consequence of encephalopathy (23, 24). Similar to our result, encephalopathy was reported as the most common neurological manifestation in many reports (14, 15, 25). Although less frequent than alteration of consciousness, neurocognitive disorders were a common CNS manifestation in our cohort, ranging from mild confusion to delirium and psychosis, and 31.9% of our CNS patients experienced this type of symptom. The postulated mechanism of these symptoms is similar to the one involved in the alteration of consciousness, but there are other factors that must be considered, such as a longer stay in an intensive care unit and prolonged administration of corticosteroids (21–24, 26).

A significant percentage (43%) of our patients presented with headaches at some point in their disease, most of them at the onset. It was most commonly described as continuous, moderately to severely intense bifrontal or bicipital pressure. One patient with no prior headache history presented with cluster-type headache. Similar to our results, most patients with COVID-19 reported tension-type or migraine-like headaches in the acute phase (27). In a meta-analysis, headache was mostly associated with non-hospitalized patients as it might be a less bothersome symptom compared to dyspnea or fever, as well as some hospitalized patients might not report it (27, 28). Furthermore, headache is also one of the common neurological manifestations associated with COVID-19. Headache reports increased during the COVID-19 pandemic, being reported in some prospective and retrospective studies in proportions ranging from 3.7 to 43% (15) The underlying mechanism for headache in COVID-19 is not fully elucidated, but there are several theories. The most commonly accepted theories postulate the direct viral invasion of the trigeminal nerve endings, hypoxemia, dehydration, endothelial dysfunction due to inflammatory process, and coagulopathies (16, 27, 28).

Acute ischemic stroke was the most common neurovascular event in our cohort, similar to other reports in the literature (29). Patients with concomitant stroke and COVID-19 were older and presented with more severe forms of infection. Nevertheless, they were younger and less likely to suffer from hypertension compared to patients with stroke in the absence of SARS-CoV-2 infection (30). Similar to data reported in the literature, in our cohort, hemorrhagic strokes were less frequent than acute ischemic stroke (29). Cerebral vein thrombosis was less frequently found as we identified it in one female patient with no previous known coagulopathy. In a study conducted by Jain et al., acute stroke was the most common COVID-19 neuroimaging finding and also a strong prognostic marker of poor outcome (31). Furthermore, SARS-CoV-2 infection is an independent risk factor for ischemic stroke according to Belani et al. (32). The mechanisms for neurovascular events remain incompletely identified, but a hypercoagulability state (high D-dimers, fibrinogen, and lupus anticoagulant), together with endothelial inflammation, is the basis for arterial and venous thrombosis associated with COVID-19 (20, 29, 30, 33).

*De novo* epileptic seizures are a less common CNS manifestation. In our cohort, we identified eight patients with seizures: six of them with motor focal seizures without generalization, one with generalized seizures, and one with reflex seizures. No underlying lesions were identified in brain imaging. Taquet et al. identified that COVID-19 was associated with a higher risk of epileptic seizures when compared with matched patients with influenza over a 6-month period from the infection (34). However, in a Swedish study performed by Westman et al., no increased risk of epilepsy was found, indicating that comorbidities may be partly responsible for the increased risk of seizures observed in COVID-19 patients, acting as confounding factors (35). The mechanism is not fully understood as it may be due to blood–brain barrier alterations secondary to inflammation that can lead to an increase in excitatory neurotransmitters, as well as ion imbalance, hypoxia, or hypoglycemia (36–38).

PNS manifestations were comprised mainly of cranial nerve involvement. All cranial nerves are susceptible, but the most affected one in our cohort was the vestibulocochlear nerve, with most patients complaining of vertigo and dizziness. Cranial nerve involvement proved to be the main peripheral nervous system manifestation also according to a systematic review of the literature (39). Anosmia was another common complaint, in 55.8% of patients. Oculomotor disturbances were found in five patients as they are a rare occurrence. In a case report and mini-review performed by Tan et al., oculomotor palsies were associated with mild COVID-19 forms and had rapid and complete recoveries (40). One patient presented with peripheral nerve palsy that remitted soon after antiviral and glucocorticoid treatment, during hospitalization, and two patients presented with polyneuritis cranialis, one of them died during hospitalization because of COVID-19 complications and the other one was discharged with sequelae. In a systematic review, the most commonly affected nerves were the facial and abducens nerves (41). The mechanism of PNS involvement continues to be a subject of debate as there are theories in favor of direct viral invasion, but inflammation also plays an important role (5, 42). Mononeuritis was identified in five patients admitted to the intensive care unit, either brachial or peroneal mononeuritis. There are a few reports in the literature, and they are attributed to vasculitis-like mechanisms of the *vasa nervorum* (43). Four patients had polyneuropathy, but because of logistic limitations during the pandemic, we were not able to assess between Guillain-Barre syndrome and other types of neuropathies, such as critical illness polyneuropathy.

Survival analysis used to evaluate the factors that are independently linked to in-hospital mortality of COVID-19 patients identified older age as a clinical marker for a poor outcome prognostic. This is valid for all COVID-19 patients, regardless of the type of neurological involvement (2). In addition, high CRP is an independent risk factor for death in patients with SARS-CoV-2 infection and associated neurological involvement. The excessive inflammation in response to the novel coronavirus is an important hallmark of the disease severity as many signs and symptoms, including neurological ones, are attributed to it (2, 15, 20)(1–3). In a systematic review performed by Silva et al., CRP together with other inflammation markers was inversely associated with neurological disease but still associated with disease severity and poor outcome (44). CRP proved to be a useful early marker to predict the risk of severe disease and death as it is closely linked to cytokine production and tissue destruction in COVID-19 patients (45, 46). Another marker that was identified as an independent risk factor for poor outcomes was the high white blood count. The *cytokine storm* exhibited a strong correlation to the white blood count (44). Furthermore, the increase in white blood count and, especially in neutrophils, correlates with disease severity and mortality in patients without proven neurological involvement (47).

Although associated with older age and severe COVID-19, involvement of the CNS is an independent risk factor for death in itself. Mao et al. observed that patients with severe illness were more likely to have CNS symptoms (3). CNS manifestations are a poor prognostic outcome, but in COVID-19, because of coagulopathy and hypoxia, patients are more prone to neurovascular involvement and encephalopathy (17, 20, 27). Because of endothelial dysfunction and associated inflammatory mechanisms, thrombectomy is more challenging and the outcome could be unfavorable (30).

Our study has several limitations. First, this is retrospective research, and not all patients who were hospitalized during this period received a complete neurological examination during their hospital stay. Therefore, the CNS and PNS involvement in SARS-CoV-2 infection could have been an underdiagnosed condition as some patients had no neurological evaluation. The lack of follow-up data after the hospitalization is another limitation as information regarding the medium- and long-term evolution of patients with neurological manifestations in COVID-19 could be valuable. The lack of data on brain imaging, electrophysiological studies, and CSF changes due to the difficulty of performing these investigations in patients with neurological deficits is another limitation of this study. In addition, we only included patients with clinical neurological manifestations, so patients with subclinical neurological involvement were overlooked.

However, in spite of the limitations, some practical messages could arise as a result of this study. Clinicians should consider the higher risk of neurological complications in patients with severe COVID-19, high D-dimers, and preexisting cardiovascular risk factors to optimize the overall management of these cases. Furthermore, patients with severe disease, associated high inflammation, and CNS involvement have a worse prognosis, and further studies addressing optimal therapeutic strategies are needed.

## 5 Conclusion

CNS manifestations are more frequent than the involvement of PNS in hospitalized patients with SARS-CoV-2 infection, and they are independently associated with older age, disease severity, ischemic heart disease, and increased D-dimers. COVID-19-associated encephalopathy was the most common CNS manifestation in our study, but neurovascular events are also important considering the overlap between inflammatory and prothrombotic pathways, especially in severe cases. PNS findings were various, involving mostly the cranial nerves, especially the vestibulocochlear nerve. Among hospitalized patients with COVID-19 older age, CNS involvement and increased level of inflammatory markers were independent risk factors for the in-hospital mortality.

## Data availability statement

The raw data supporting the conclusions of this article will be made available by the authors, without undue reservation.

## Ethics statement

The studies involving humans were approved by the Ethics Committee of the National Institute of Infectious Diseases “Matei Bals”. The studies were conducted in accordance with the local legislation and institutional requirements. The human samples used in this study were acquired from a by- product of routine care or industry. Written informed consent for participation was not required from the participants or the participants’ legal guardians/next of kin in accordance with the national legislation and institutional requirements.

## Author contributions

AnH: Conceptualization, Data curation, formal analysis, Investigation, Methodology, Writing – original draft, Writing – review & editing. C-MN: Conceptualization, Data curation, Methodology, Writing – original draft, Writing – review & editing. AD: Conceptualization, Supervision, Writing – review & editing, Validation. EM: Conceptualization, Formal Analysis, Methodology, Writing – review & editing, Validation. RC: Data curation, Methodology, Writing – original draft. GN: Data curation, Methodology, Writing – original draft. AdH: Conceptualization, Methodology, Project administration, Supervision, Writing – review & editing, Validation.

## References

[1] Gudowska-Sawczuk M Mroczko B. The role of Neuropilin-1 (NRP-1) in SARS-CoV-2 infection: review. J Clin Med. (2021) 10:2772. doi: 10.3390/jcm10132772, PMID: 34202613 PMC8267897

[2] Zhou F Yu T Du R Fan G Liu Y Liu Z. Clinical course and risk factors for mortality of adult inpatients with COVID-19 in Wuhan, China: a retrospective cohort study. Lancet. (2020) 395:1054–62. doi: 10.1016/S0140-6736(20)30566-3, PMID: 32171076 PMC7270627

[3] Mao L Jin H Wang M Hu Y Chen S He Q. Neurologic manifestations of hospitalized patients with coronavirus disease 2019 in Wuhan, China. JAMA Neurol. (2020) 77:683. doi: 10.1001/jamaneurol.2020.1127, PMID: 32275288 PMC7149362

[4] Gao Y Chen Y Liu M Niu M Song Z Yan M. Nervous system diseases are associated with the severity and mortality of patients with COVID-19: a systematic review and meta-analysis. Epidemiol Infect. (2021) 149:e66. doi: 10.1017/S0950268821000376, PMID: 33583450 PMC7985867

[5] Harapan BN Yoo HJ. Neurological symptoms, manifestations, and complications associated with severe acute respiratory syndrome coronavirus 2 (SARS-CoV-2) and coronavirus disease 19 (COVID-19). J Neurol. (2021) 268:3059–71. doi: 10.1007/s00415-021-10406-y, PMID: 33486564 PMC7826147

[6] Spudich S Nath A. Nervous system consequences of COVID-19. Science (1979). (2022) 375:267–9. doi: 10.1126/science.abm2052 35050660

[7] Balloff C Bandlow C Bernhard M Brandenburger T Bludau P Elben S. Prevalence and prognostic value of neurological affections in hospitalized patients with moderate to severe COVID-19 based on objective assessments. Sci Rep. (2023) 13:19619. doi: 10.1038/s41598-023-46124-w, PMID: 37949882 PMC10638293

[8] Cho SM White N Premraj L Battaglini D Fanning J Suen J. Neurological manifestations of COVID-19 in adults and children. Brain. (2023) 146:1648–61. doi: 10.1093/brain/awac332, PMID: 36087305 PMC9494397

[9] Singh B Lant S Cividini S Cattrall JWS Goodwin LC Benjamin L. Prognostic indicators and outcomes of hospitalised COVID-19 patients with neurological disease: an individual patient data meta-analysis. PLoS One. (2022) 17:e0263595. doi: 10.1371/journal.pone.0263595, PMID: 35653330 PMC9162376

[10] Siahaan YMT Puspitasari V Pangestu A. COVID-19-associated encephalopathy: systematic review of case reports. J Clin Neurol. (2022) 18:194. doi: 10.3988/jcn.2022.18.2.194, PMID: 35196749 PMC8926776

[11] Daripa B Lucchese S. Unusual presentation of COVID-19 headache and its possible Pathomechanism. Cureus. (2022) 14:e29358. doi: 10.7759/cureus.29358, PMID: 36284805 PMC9583707

[12] Tana C Bentivegna E Cho SJ Harriott AM García-Azorín D Labastida-Ramirez A. Long COVID headache. J Headache Pain. (2022) 23:93. doi: 10.1186/s10194-022-01450-8, PMID: 35915417 PMC9340759

[13] Niculae CM Anghel AMJ Militaru ED Tîrlescu LG Lazar M Hristea A. Acute pulmonary artery thrombosis despite anticoagulation in patients with COVID-19 pneumonia: a single-center retrospective cohort study. J Clin Med. (2022) 11:2633. doi: 10.3390/jcm11092633, PMID: 35566758 PMC9100155

[14] Nersesjan V Amiri M Lebech AM Roed C Mens H Russell L. Central and peripheral nervous system complications of COVID-19: a prospective tertiary center cohort with 3-month follow-up. J Neurol. (2021) 268:3086–104. doi: 10.1007/s00415-020-10380-x, PMID: 33438076 PMC7803470

[15] Ousseiran ZH Fares Y Chamoun WT. Neurological manifestations of COVID-19: a systematic review and detailed comprehension. Int J Neurosci. (2023) 133:754–69. doi: 10.1080/00207454.2021.1973000, PMID: 34433369 PMC8506813

[16] Bolay H Gül A Baykan B. COVID-19 is a real headache! Headache: the journal of head and face. Pain. (2020) 60:1415–21. doi: 10.1111/head.13856 PMC7272895 32412101

[17] Cui Y Zhao B Li T Yang Z Li S Le W. Risk of ischemic stroke in patients with COVID-19 infection: a systematic review and meta-analysis. Brain Res Bull. (2022) 180:31–7. doi: 10.1016/j.brainresbull.2021.12.011, PMID: 34979237 PMC8719366

[18] Nemec HM Ferenczy A Christie BD Ashley DW Montgomery A. Correlation of D-dimer and outcomes in COVID-19 patients. Am Surg. (2022) 88:2115–8. doi: 10.1177/00031348221091940, PMID: 35487527 PMC9066233

[19] Liang C Zhang W Li S Qin G. Coronary heart disease and COVID-19: a meta-analysis. Med Clin (Barc). (2021) 156:547–54. doi: 10.1016/j.medcli.2020.12.017, PMID: 33632508 PMC7843088

[20] Varga Z Flammer AJ Steiger P Haberecker M Andermatt R Zinkernagel AS. Endothelial cell infection and endotheliitis in COVID-19. Lancet. (2020) 395:1417–8. doi: 10.1016/S0140-6736(20)30937-5, PMID: 32325026 PMC7172722

[21] Solomon IH Normandin E Bhattacharyya S Mukerji SS Keller K Ali AS. Neuropathological features of Covid-19. N Engl J Med. (2020) 383:989–92. doi: 10.1056/NEJMc2019373, PMID: 32530583 PMC7304421

[22] Thakur KT Miller EH Glendinning MD Al-Dalahmah O Banu MA Boehme AK. COVID-19 neuropathology at Columbia University Irving medical center/New York Presbyterian hospital. Brain. (2021) 144:2696–708. doi: 10.1093/brain/awab148, PMID: 33856027 PMC8083258

[23] Iadecola C Anrather J Kamel H. Effects of COVID-19 on the nervous system. Cells. (2020) 183:16–27.e1. doi: 10.1016/j.cell.2020.08.028, PMID: 32882182 PMC7437501

[24] Huang YH Jiang D Huang JT. SARS-CoV-2 detected in cerebrospinal fluid by PCR in a case of COVID-19 encephalitis. Brain Behav Immun. (2020) 87:149. doi: 10.1016/j.bbi.2020.05.012, PMID: 32387508 PMC7202824

[25] Liguori C Pierantozzi M Spanetta M Sarmati L Cesta N Iannetta M. Subjective neurological symptoms frequently occur in patients with SARS-CoV2 infection. Brain Behav Immun. (2020) 88:11–6. doi: 10.1016/j.bbi.2020.05.037, PMID: 32416289 PMC7235586

[26] Martinotti G Bonanni L Barlati S Miuli A Sepede G Prestia D. Delirium in COVID-19 patients: a multicentric observational study in Italy. Neurol Sci. (2021) 42:3981–8. doi: 10.1007/s10072-021-05461-2, PMID: 34318364 PMC8316107

[27] Sampaio Rocha-Filho PA. Headache associated with COVID-19: epidemiology, characteristics, pathophysiology, and management. Headache: The J Head and Face Pain. (2022) 62:650–6. doi: 10.1111/head.14319 PMC9348060 35545780

[28] Fernández-de-las-Peñas C Navarro-Santana M Gómez-Mayordomo V Cuadrado ML García-Azorín D Arendt-Nielsen L. Headache as an acute and post-COVID-19 symptom in COVID-19 survivors: a meta-analysis of the current literature. Eur J Neurol. (2021) 28:3820–5. doi: 10.1111/ene.15040, PMID: 34327787 PMC8444899

[29] Nannoni S de Groot R Bell S Markus HS. Stroke in COVID-19: a systematic review and meta-analysis. Int J Stroke. (2021) 16:137–49. doi: 10.1177/1747493020972922, PMID: 33103610 PMC7859578

[30] Wang A Mandigo GK Yim PD Meyers PM Lavine SD. Stroke and mechanical thrombectomy in patients with COVID-19: technical observations and patient characteristics. J Neurointerv Surg. (2020) 12:648–53. doi: 10.1136/neurintsurg-2020-016220, PMID: 32451359

[31] Jain R Young M Dogra S Kennedy H Nguyen V Jones S. COVID-19 related neuroimaging findings: a signal of thromboembolic complications and a strong prognostic marker of poor patient outcome. J Neurol Sci. (2020) 414:116923. doi: 10.1016/j.jns.2020.116923, PMID: 32447193 PMC7236667

[32] Belani P Schefflein J Kihira S Rigney B Delman BN Mahmoudi K. COVID-19 is an independent risk factor for acute ischemic stroke. Am J Neuroradiol. (2020) 41:1361–4. doi: 10.3174/ajnr.A6650, PMID: 32586968 PMC7658882

[33] Bowles L Platton S Yartey N Dave M Lee K Hart DP. Lupus anticoagulant and abnormal coagulation tests in patients with Covid-19. N Engl J Med. (2020) 383:288–90. doi: 10.1056/NEJMc2013656, PMID: 32369280 PMC7217555

[34] Taquet M Devinsky O Cross JH Harrison PJ Sen A. Incidence of epilepsy and seizures over the first 6 months after a COVID-19 diagnosis. Neurology. (2023) 100:e790–9. doi: 10.1212/WNL.0000000000201595, PMID: 36384658 PMC9984208

[35] Westman G Zelano J. Epilepsy diagnosis after Covid-19: a population-wide study. Seizure: European. J Epilepsy. (2022) 101:11–4. doi: 10.1016/j.seizure.2022.07.005 PMC9270960 35842976

[36] Boz PB Aslan-Kara K Şanlı ZS Peköz MT Acar D Bozdemir H. Seizures in COVID-19: the relationship between biomarkers and prognosis. Acta Neurol Belg. (2023) 123:1763–72. doi: 10.1007/s13760-022-02054-4, PMID: 35907150 PMC9362485

[37] Riazi K Galic MA Pittman QJ. Contributions of peripheral inflammation to seizure susceptibility: cytokines and brain excitability. Epilepsy Res. (2010) 89:34–42. doi: 10.1016/j.eplepsyres.2009.09.004, PMID: 19804959

[38] Nikbakht F Mohammadkhanizadeh A Mohammadi E. How does the COVID-19 cause seizure and epilepsy in patients? The potential mechanisms. Mult Scler Relat Disord. (2020) 46:102535. doi: 10.1016/j.msard.2020.102535, PMID: 33010584 PMC7521932

[39] Hanganu AR Constantin A Moise ES Niculae CM Olaru ID Băicuș C. Peripheral nervous system involvement associated with COVID-19. A systematic review of literature. PLoS One. (2023) 18:e0283827. doi: 10.1371/journal.pone.0283827, PMID: 37023030 PMC10079054

[40] Tan YJ Ramesh R Tan YH Tan SML Setiawan S. COVID-19 and isolated oculomotor nerve palsy: clinical features and outcomes. Clin Neurol Neurosurg. (2023) 225:107601. doi: 10.1016/j.clineuro.2023.107601, PMID: 36696848 PMC9850642

[41] Tonkal A Alamri AA AlMaghrabi SJ Mozahim NF Mozahim SF Alsubaie SA. Cranial nerve impairment associated with COVID-19 infections: a systematic review. Cureus. (2022) 14:e31997. doi: 10.7759/cureus.31997, PMID: 36589199 PMC9798034

[42] Mahboubi Mehrabani M Karvandi MS Maafi P Doroudian M. Neurological complications associated with Covid-19; molecular mechanisms and therapeutic approaches. Rev Med Virol. (2022) 32:e2334. doi: 10.1002/rmv.2334, PMID: 35138001 PMC9111040

[43] Acharya S Thibault M Lee J Taha O Morpurgo AJ Kshetree BK. COVID-19-induced left sciatic neuropathy requiring prolonged physical medicine and rehabilitation. Cureus. (2021) 13:e15803. doi: 10.7759/cureus.15803, PMID: 34306871 PMC8294023

[44] Silva MJA Ribeiro LR Gouveia MIM Dos MBR Dos SCS KVB L. Hyperinflammatory response in COVID-19: a systematic review. Viruses. (2023) 15:553. doi: 10.3390/v15020553, PMID: 36851766 PMC9962879

[45] Hachim IY Hachim MY Hannawi H Bin NK Salah A Hannawi S. The inflammatory biomarkers profile of hospitalized patients with COVID-19 and its association with patient’s outcome: a single centered study. PLoS One. (2021) 16:e0260537. doi: 10.1371/journal.pone.0260537, PMID: 34855832 PMC8638892

[46] Smilowitz NR Kunichoff D Garshick M Shah B Pillinger M Hochman JS. C-reactive protein and clinical outcomes in patients with COVID-19. Eur Heart J. (2021) 42:2270–9. doi: 10.1093/eurheartj/ehaa1103, PMID: 33448289 PMC7928982

[47] He R Lu Z Zhang L Fan T Xiong R Shen X. The clinical course and its correlated immune status in COVID-19 pneumonia. J Clin Virol. (2020) 127:104361. doi: 10.1016/j.jcv.2020.104361, PMID: 32344320 PMC7152870

