# Supplementary material for: The outcome and risk factors associated with central and peripheral nervous system involvement in hospitalized COVID-19 patients: a retrospective cohort study
Source: Front Neurol. 2024 Jan 11;14:1338593. doi: 10.3389/fneur.2023.1338593 (PMC10808716; doi:10.3389/fneur.2023.1338593)
Supplement: Supplementary file 1 [file Data_Sheet_1.PDF]

```
COXREG durata_internarezile
/STATUS=Outcome(2)
/CONTRAST (Remdesivir)=Indicator
/CONTRAST (DZ)=Indicator
/CONTRAST (sex)=Indicator
/CONTRAST (obezitate)=Indicator
/CONTRAST (formaCOVID)=Indicator
/CONTRAST (SNCSNP)=Indicator
/CONTRAST (Dexametzon)=Indicator
/CONTRAST (Bcard)=Indicator
/CONTRAST (Imunoterapie)=Indicator
/CONTRAST (Antiagregant)=Indicator
/CONTRAST (AVC_YN)=Indicator
/CONTRAST (BRC)=Indicator
/METHOD=FSTEP(LR) varsta_debut sex DZ obezitate Creatinina CRP Uree SNCSNP
Dexametzon Bcard
Imunoterapie Remdesivir BRC formaCOVID AVC_YN Anticoagulant Antiagregant WBC
/PRINT=CI(95) SUMMARY
/CRITERIA=PIN(.05) POUT(.10) ITERATE(20).
```

Cox Regression

Notes

Output Created		08-AUG-2023 20:41:...
Comments		
Input	Data	C:\Users\Emi\Desktop\studiu covid andreea florea\neurocovid_database 1 (1).sav
	Active Dataset	DataSet1
	Filter	<none>
	Weight	<none>
	Split File	<none>
	N of Rows in Working Data File	115
Missing Value Handling	Definition of Missing	User-defined missing values are treated as missing.

Notes

Syntax		COXREG durata_internarezile<br>/STATUS=Outcome(2)<br>/CONTRAST (Remdesivir)=Indicator<br>/CONTRAST (DZ)=Indicator<br>/CONTRAST (sex)=Indicator<br>/CONTRAST (obezitate)=Indicator<br>/CONTRAST (formaCOVID)=Indicator<br>/CONTRAST (SNCSNP)=Indicator<br>/CONTRAST (Dexametzon)=Indicator<br>/CONTRAST (Bcard)=Indicator<br>/CONTRAST (Imunoterapie)=Indicator<br>/CONTRAST (Antiagregant)=Indicator<br>/CONTRAST (AVC_YN)=Indicator<br>/CONTRAST (BRC)=Indicator<br>/METHOD=FSTEP(LR) varsta_debut sex DZ obezitate Creatinina CRP Uree SNCSNP Dexametzon Bcard Imunoterapie Remdesivir BRC formaCOVID AVC_YN Anticoagulant Antiagregant WBC<br>/PRINT=CI(95) SUMMARY<br>/CRITERIA=PIN(.05) POUT(.10) ITERATE(20).
Resources	Processor Time	00:00:00.06
	Elapsed Time	00:00:00.02

Case Processing Summary

		N	Percent
Cases available in analysis	Event[a]	24	20,9%
	Censored	84	73,0%
	Total	108	93,9%
Cases dropped	Cases with missing values	2	1,7%
	Cases with negative time	0	0,0%
	Censored cases before the earliest event in a stratum	5	4,3%
	Total	7	6,1%
Total		115	100,0%

a. Dependent Variable: durata_internare (zile)

Categorical Variable Codings[a,c,d,f,g,h,i,j,k,l,m,n]

		Frequency	(1)[e]
sex[b]	1	53	1
	2	60	0
forma COVID[b]	1	52	1
	2	61	0
DZ[b]	0	73	1
	1	40	0
Bcard[b]	,00	45	1
	1,00	68	0
obezitate[b]	0	81	1
	1	32	0
BRC[b]	0	106	1
	1	7	0
AVC_YN[b]	0	93	1
	1	20	0
SNC/SNP[b]	1	72	1
	2	41	0
Imunoterapie[b]	0	67	1
	1	46	0
Dexametzon[b]	0	20	1
	1	93	0
Antiagregant[b]	0	104	1
	1	9	0
Remdesivir[b]	0	59	1
	1	54	0

a. Category variable: sex

b. Indicator Parameter Coding

c. Category variable: forma COVID (formaCOVID)

d. Category variable: DZ

e. The (0,1) variable has been recoded, so its coefficients will not be the same as for indicator (0,1) coding.

f. Category variable: Bcard

g. Category variable: obezitate

h. Category variable: BRC

i. Category variable: AVC_YN

j. Category variable: SNC/SNP (SNCSNP)

k. Category variable: Imunoterapie (Imunoterapie)

l. Category variable: Dexametzon

m. Category variable: Antiagregant

n. Category variable: Remdesivir

Block 0: Beginning Block

Omnibus Tests of Model Coefficients

-2 Log Likelihood
168,039

Block 1: Method = Forward Stepwise (Likelihood Ratio)

Omnibus Tests of Model Coefficients[a]

Step	-2 Log Likelihood	Overall (score)			Change From Previous Block		
		Chi-square	df	Sig.	Chi-square	df	Sig.
4	131,323	41,665	4	,000	36,716	4	,000

a. Beginning Block Number 1. Method = Forward Stepwise (Likelihood Ratio)

Variables in the Equation

		B	SE	Wald	df	Sig.	Exp(B)
Step 4	varsta_debut	,058	,017	11,065	1	,001	1,059
	CRP	,006	,003	4,592	1	,032	1,006
	SNC/SNP	2,214	1,043	4,506	1	,034	9,155
	WBC	,052	,013	14,864	1	,000	1,053

Variables in the Equation

		95.0% CI for Exp(B)	
		Lower	Upper
Step 4	varsta_debut	1,024	1,096
	CRP	1,000	1,011
	SNC/SNP	1,185	70,738
	WBC	1,026	1,081

Variables not in the Equation[a]

		Score	df	Sig.
Step 4	sex	1,265	1	,261
	DZ	,005	1	,944
	obezitate	2,414	1	,120
	Creatinina	,187	1	,666
	Uree	,128	1	,721
	Dexametzon	1,656	1	,198
	Bcard	,529	1	,467
	Imunoterapie	1,470	1	,225
	Remdesivir	,049	1	,826
	BRC	1,673	1	,196
	forma COVID	1,491	1	,222
	AVC_YN	,646	1	,422
	Anticoagulant	,071	1	,789
	Antiagregant	1,210	1	,271

a. Residual Chi Square = 16.651 with 14 df Sig. = .275

Covariate Means

	Mean
varsta_debut	61,898
sex	,481
DZ	,639
obezitate	,713
Creatinina	1,056
CRP	81,499
Uree	60,298
SNC/SNP	,630
Dexametzon	,167
Bcard	,398
Imunoterapie	,593
Remdesivir	,537
BRC	,935
forma COVID	,444
AVC_YN	,833
Anticoagulant	1,361
Antiagregant	,917
WBC	10,061

```
COXREG debut_neurozile
/STATUS=SNCSNP(1)
/CONTRAST (Remdesivir)=Indicator
/CONTRAST (DZ)=Indicator
/CONTRAST (sex)=Indicator
/CONTRAST (obezitate)=Indicator
/CONTRAST (formaCOVID)=Indicator
/CONTRAST (Dexametzon)=Indicator
/CONTRAST (Bcard)=Indicator
/CONTRAST (Imunoterapie)=Indicator
/CONTRAST (Antiagregant)=Indicator
/CONTRAST (BRC)=Indicator
/METHOD=FSTEP(LR) varsta_debut sex DZ obezitate Creatinina CRP Uree Dexametzon Bcard
Imunoterapie Remdesivir formaCOVID Anticoagulant Antiagregant WBC Limfocite HTA FIA
Insuf_cardiaca
BCI Fumtor Etanol BRC Ddimeri
/PRINT=CI(95) SUMMARY
/CRITERIA=PIN(.05) POUT(.10) ITERATE(20).
```

Cox Regression

Notes

Output Created		08-AUG-2023 20:50:...
Comments		
Input	Data	C: \Users\Emi\Desktop\studiu covid andreea florea\neurocovid_database 1 (1).sav
	Active Dataset	DataSet1
	Filter	<none>
	Weight	<none>
	Split File	<none>
	N of Rows in Working Data File	115
Missing Value Handling	Definition of Missing	User-defined missing values are treated as missing.
Syntax		COXREG debut_neurozile<br>/STATUS=SNCSNP(1)<br>/CONTRAST (Remdesivir)=Indicator<br>/CONTRAST (DZ)=Indicator<br>/CONTRAST (sex)=Indicator<br>/CONTRAST (obezitate)=Indicator<br>/CONTRAST (formaCOVID)=Indicator<br>/CONTRAST (Dexametzon)=Indicator<br>/CONTRAST (Bcard)=Indicator<br>/CONTRAST (Imunoterapie)=Indicator<br>/CONTRAST (Antiagregant)=Indicator<br>/CONTRAST (BRC)=Indicator<br>/METHOD=FSTEP(LR) varsta_debut sex DZ obezitate Creatinina CRP Uree Dexametzon Bcard<br>Imunoterapie Remdesivir formaCOVID Anticoagulant Antiagregant WBC Limfocite HTA FIA Insuf_cardiaca<br>BCI Fumtor Etanol BRC Ddimeri<br>/PRINT=CI(95) SUMMARY<br>/CRITERIA=PIN(.05) POUT(.10) ITERATE(20).

Notes

Resources	Processor Time	00:00:00.03
	Elapsed Time	00:00:00.03

Case Processing Summary

		N	Percent
Cases available in analysis	Event[a]	68	59,1%
	Censored	37	32,2%
	Total	105	91,3%
Cases dropped	Cases with missing values	10	8,7%
	Cases with negative time	0	0,0%
	Censored cases before the earliest event in a stratum	0	0,0%
	Total	10	8,7%
Total		115	100,0%

a. Dependent Variable: debut_neuro (zile)

Categorical Variable Codings[a,c,d,f,g,h,i,j,k,l]

		Frequency	(1)[e]
sex[b]	1	50	1
	2	55	0
forma COVID[b]	1	48	1
	2	57	0
DZ[b]	0	66	1
	1	39	0
Bcard[b]	,00	41	1
	1,00	64	0
obezitate[b]	0	77	1
	1	28	0
BRC[b]	0	99	1
	1	6	0
Imunoterapie[b]	0	61	1
	1	44	0
Dexametzon[b]	0	19	1
	1	86	0
Antiagregant[b]	0	96	1
	1	9	0
Remdesivir[b]	0	55	1
	1	50	0

a. Category variable: sex

b. Indicator Parameter Coding

c. Category variable: forma COVID (formaCOVID)

d. Category variable: DZ

e. The (0,1) variable has been recoded, so its coefficients will not be the same as for indicator (0,1) coding.

f. Category variable: Bcard

g. Category variable: obezitate

h. Category variable: BRC

i. Category variable: Imunoterapie (Imunoterapie)

j. Category variable: Dexametzon

k. Category variable: Antiagregant

l. Category variable: Remdesivir

Block 0: Beginning Block

Omnibus Tests of Model Coefficients

-2 Log Likelihood
497,875

Block 1: Method = Forward Stepwise (Likelihood Ratio)

Omnibus Tests of Model Coefficients[a]

Step	-2 Log Likelihood	Overall (score)			Change From Previous Block		
		Chi-square	df	Sig.	Chi-square	df	Sig.
4	477,175	25,115	4	,000	20,700	4	,000

a. Beginning Block Number 1. Method = Forward Stepwise (Likelihood Ratio)

Variables in the Equation

		B	SE	Wald	df	Sig.	Exp(B)
Step 4	varsta_debut	,020	,008	5,576	1	,018	1,020
	forma COVID	,929	,294	9,988	1	,002	2,532
	BCI	,886	,427	4,305	1	,038	2,426
	D-dimeri	,000	,000	5,410	1	,020	1,000

Variables in the Equation

		95.0% CI for Exp(B)	
		Lower	Upper
Step 4	varsta_debut	1,003	1,037
	forma COVID	1,423	4,505
	BCI	1,050	5,602
	D-dimeri	1,000	1,000

Variables not in the Equation[a]

		Score	df	Sig.
Step 4	sex	,477	1	,490
	DZ	1,030	1	,310
	obezitate	,625	1	,429
	Creatinina	1,388	1	,239
	CRP	,057	1	,812
	Uree	1,149	1	,284
	Dexametzon	,911	1	,340
	Bcard	,740	1	,390
	Imunoterapie	,607	1	,436
	Remdesivir	,055	1	,815
	Anticoagulant	,547	1	,459
	Antiagregant	,667	1	,414
	WBC	1,282	1	,257
	Limfocite	,030	1	,861
	HTA	,131	1	,717
	FIA	2,235	1	,135
	Insuf_cardiaca	,136	1	,713
	Fumtor	,064	1	,800
	Etanol	,008	1	,929
	BRC	,029	1	,865

a. Residual Chi Square = 19.682 with 20 df Sig. = .478

Covariate Means

	Mean
varsta_debut	62,686
sex	,476
DZ	,629
obezitate	,733
Creatinina	1,014
CRP	80,022
Uree	59,230
Dexametzon	,181
Bcard	,390
Imunoterapie	,581
Remdesivir	,524
forma COVID	,457
Anticoagulant	1,371
Antiagregant	,914
WBC	10,012
Limfocite	1,053
HTA	,581
FIA	,171
Insuf_cardiaca	,105
BCI	,086
Fumtor	,038
Etanol	,010
BRC	,943
D-dimeri	828,667
